# Understanding Protein and Polysaccharide Fouling with Silicon Dioxide and Aluminum Oxide in Low-Pressure Membranes

**DOI:** 10.3390/membranes13050476

**Published:** 2023-04-28

**Authors:** Mohammad T. Alresheedi

**Affiliations:** Department of Civil Engineering, College of Engineering, Qassim University, Buraydah 51452, Saudi Arabia; m.alresheedi@qu.edu.sa

**Keywords:** water treatment, ultrafiltration membrane, protein fouling, polysaccharide fouling, colloidal silica fouling, α-aluminum oxide fouling

## Abstract

Humic, protein, and polysaccharide substances have been recognized as significant types of foulants in membrane systems. Despite the remarkable amount of research that has been performed on the interaction of these foulants, particularly humic and polysaccharide substances, with inorganic colloids in RO systems, little attention has been paid to the fouling and cleaning behavior of proteins with inorganic colloids in UF membranes. This research examined the fouling and cleaning behavior of bovine serum albumin (BSA) and sodium alginate (SA) with silicon dioxide (SiO_2_) and α-aluminum oxide (Al_2_O_3_) in individual and combined solutions during dead-end UF filtration. The results showed that the presence of SiO_2_ or Al_2_O_3_ in water alone did not cause significant fouling or a flux decline in the UF system. However, the combination of BSA and SA with inorganics was observed to have a synergistic effect on membrane fouling, in which the combined foulants caused higher irreversibility than individual foulants. Analysis of blocking laws demonstrated that the fouling mechanism shifted from cake filtration to complete pore blocking when the combined organics and inorganics were present in water, which resulted in higher BSA and SA fouling irreversibility. The results suggest that membrane backwash needs to be carefully designed and adjusted for better control of BSA and SA fouling with SiO_2_ and Al_2_O_3_.

## 1. Introduction

The application of UF membranes in the production of drinking water has been increasing over the past few decades. However, membrane fouling due to the accumulation of water constituents such as organic and inorganic colloids on the membrane surface or within pores remains a challenge for maintaining the good performance of UF membranes. Once fouling occurs, it reduces membrane flux and increases the pressure required to maintain membrane productivity. Moreover, membrane fouling increases the need for physical backwashing, chemical cleaning, and membrane replacement. While membrane fouling has been extensively researched in the past, studies on how protein and polysaccharide substances with inorganic colloids interact and affect the fouling characteristics of low-pressure UF membranes are limited.

Few studies exist that have studied the fouling behavior of organics and inorganics, most of which have high-pressure reverse osmosis (RO) membranes in water desalination. Melián-Martel et al. [1] investigated the fouling behavior of RO membranes subjected to fouling by SA and colloidal silica in single and combined feed solutions. The results showed an elevated level of fouling and higher flux decline when a combined solution was filtered compared to a solution with single foulants. In another study, Wang et al. [2] investigated the fouling of SA and sodium silicate during the RO process. Membrane fouling was the highest during the filtration of combined silicate and SA solutions, suggesting a synergistic effect between the two foulants. Few papers examined SA fouling in the presence of other inorganic compounds such as iron (Fe^2+^), magnesium (Mg^2+^), and calcium (Ca^2+^). Xin et al. [3] investigated the fouling behavior of SA and Fe^2+^ with and without Ca^2+^. Their study found that the cake layer produced by the Fe^2+^- SA had higher resistance than that produced by the Ca^2+^- SA. However, it was also found that SA concentration is an important parameter that determines the properties of the Fe^2+^- SA fouling layers. Fe^2+^- SA fouling increased when Ca^2+^, which was present due to the less-permeable cake layer, formed on the membrane. The effect of Ca^2+^ on SA fouling was also demonstrated in other studies [3,4,5]. Zhang et al. [5] examined the effect of Ca^2+^ on SA fouling and found that the alginate structure and fouling formation changed as the Ca^2+^ concentration increased in the solution. Wang et al. [6] investigated the mechanism of SA fouling in the presence of Mg^2+^. Their results showed an increase in SA fouling with an increase in Mg^2+^ concentration in the solution. Gel layer formation was the dominant fouling mechanism under all tested conditions due to the interaction of alginate molecules with Mg^2+^. Charfi et al. [7] examined the fouling of SA at different concentrations with the addition of cations (Na^+^ and Ca^2+^) during microfiltration. The addition of both cations increased the amount of fouling and resulted in a high-density cake layer on the membrane.

While the above studies addressed the fouling mechanism of SA, other types of organic matter, such as proteins, may present in water and lead to membrane fouling as well. For instance, researchers [8,9,10,11] studied the fouling behavior of organics alone in mixed and individual solutions. Hashino et al. [8] reported that during the filtration of a BSA solution, flux decline was more pronounced compared to that with a humic acid, which is possibly due to the stronger adsorption of BSA on the membrane surface. Similar observations were reported by [11] with regard to the fouling of a BSA–SA mixture under constant flux. In their study, the fouling of the mixture was significantly increased compared to that with single foulants. In another study, Alresheedi et al. [12] demonstrated a higher fouling index and irreversibility of BSA compared to SA and humic acid, which influenced membrane cleaning. Thus, the type of organics in water plays an important role in membrane fouling and cleaning.

As presented above, although there has been a remarkable amount of research carried out on organic fouling with inorganic substances, particularly involving SA with Ca^2+^, studies on low-pressure UF membrane fouling by proteins in a combined mixture with inorganic colloids are much less common, which has had a negative impact on the design and operation of UF systems. In addition, many previous works on organic and inorganic fouling are limited to high-pressure RO membrane systems, which have significantly different feed water composition, membrane configuration, and operating condition than low-pressure UF systems. Therefore, the objective of this research was to investigate the fouling and cleaning behavior of BSA versus SA with and without SiO_2_ and α-Al_2_O_3_ during low-pressure dead-end UF membrane filtration. The specific objectives are: (1) to investigate BSA, SA, SiO_2_, and α-Al_2_O_3_ fouling alone and in combined solutions in a UF membrane system; (2) to examine the effect of SiO_2_ and α-Al_2_O_3_ on the rejection, cake layer properties, and fouling mechanism of BSA and SA; and (3) to assess membrane backwash effectiveness during the filtration of individual and combined organic and inorganic solutions. The results of the present study can help us gain a deeper understanding of UF membrane fouling by BSA versus SA in the presence and absence of SiO_2_ and α-Al_2_O_3_ and their impact on membrane backwash efficacy, allowing us to better predict potential changes in membrane cleaning requirements.

## 2. Materials and Methods

### 2.1. Feed Water and Model Foulants

Feed water was prepared to mimic the concentration of foulants in natural surface waters. BSA (Sigma-Aldrich, Waltham, MA, USA) and SA (Sigma-Aldrich) were chosen as the representatives of proteins and polysaccharides in water [12]. SiO_2_ (Sigma-Aldrich) and α-Al_2_O_3_ (Sigma-Aldrich) were chosen to represent model inorganic foulants in natural waters [13].

The concentration of foulants used in the experiments was 10 mg/L for BSA and SA and 100 mg/L for SiO_2_ and Al_2_O_3_. The ionic strength of the synthetic feed water solutions was adjusted by adding sodium chloride (NaCl, 2 mM) and calcium chloride (CaCl_2_, 1.5 mM). The pH of all solutions was adjusted as needed to 7.0 ± 0.2. Synthetic feed solutions were prepared by adding model foulants to deionized water (DI) and were mixed for at least 3 h prior to filtration. For all experiments, the permeability decline during filtration (i.e., actual fouling) was compared to the theoretical permeability by adding the organic and inorganic permeability declines (i.e., simple addition). The conducted fouling experiments with the individual and combined solutions are summarized and presented in Table 1.

### 2.2. Experimental Setup and Filtration Conditions

**Figure 1:**
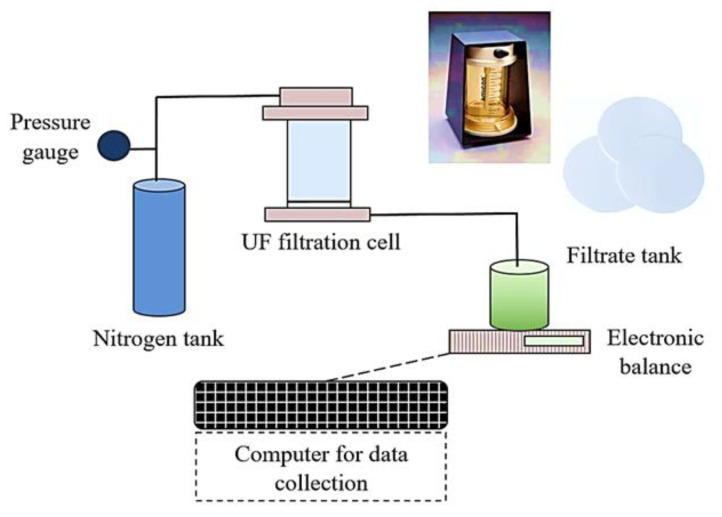
Schematic representation of the UF filtration setup.

Fouling experiments were conducted using a lab-scale filtration setup (Figure 1). The filtration setup consisted of Amicon 8400 stirred filtration cells (Millipore, Burlington, MA, USA) at a volume of 400 mL and flat-sheet, negatively charged, regenerated cellulose UF membranes with the following characteristics: pore size of 0.04 µm, molecular weight cut-off (MWCO) of 100 kDa, and diameter of 76 mm. Filtrate was collected in a beaker on an electric balance which was connected to a computer in order to record the filtrate volume and filtration time data every 30 s. Feed water was pressurized by nitrogen gas for filtration.

The resistance-in-series model was applied to determine membrane and fouling resistances. Briefly, prior to use, UF membranes were soaked in pure milli-Q water for 24 h before being used and stored at 4 °C. Before the start of the experiments, membranes were flushed with milli-Q water at room temperature to remove any organic residues. New membranes were used for the fouling tests. The intrinsic membrane resistance was determined by filtering 300 mL of milli-Q water through the membrane at a constant pressure of 1 bar (15 psi). Subsequently, the milli-Q water in the filtration cell was then replaced with synthetic feed solutions containing foulants. Fouling experiments were performed at a constant pressure of 1 bar, which was applied with the nitrogen gas in dead-end mode. Fouling experiments continued until a specific permeate volume (300 mL) had been collected. The backwashing of UF membranes was then performed by turning the filter upside down and using 200 mL of the permeate water under an applied pressure of 1.5 bar (1.5× higher than the filtration pressure), which was applied by the nitrogen gas. Finally, 300 mL of milli-Q water was then filtered to determine the reversible and irreversible fouling resistances after backwash.

Fouling resistances during the experiments were obtained using Equations (1)–(3) [14].

(1) $$R_{m}=\frac{\Delta P}{\mu J_{0}}$$

(2) $$R_{\mathrm{rev}}=\frac{\Delta P}{\mu J_{w}}-\frac{\Delta P}{\mu J_{f}}$$

(3) $$R_{\mathrm{irr}}=\frac{\Delta P}{\mu J_{w}}-\frac{\Delta P}{\mu J_{0}}$$

where R_m_, R_rev_, and R_irr_ refer to intrinsic membrane, reversible, and irreversible resistances, respectively (m^−1^). J_0_ is the initial flux (m/s). J_f_ is the final flux at the end of the fouling experiments (m/s). J_w_ is the water flux after backwash (m/s). ΔP is the filtration pressure (Pa). µ is the water viscosity (Pa s).

### 2.3. Analytical Methods

The surface changes in the membrane due to fouling were evaluated by estimating the specific cake resistance and cake compressibility index of the fouling layer. The cake layer resistance (R_c_) is a function of the specific cake resistance, α_c_ (m/g), the particle concentration, C_b_ (kg/m^3^), and the water volume, V_s_ (m^3^/m^2^), as shown in Equation (4).

(4) $$R_{c}=\alpha_{c}C_{b}V_{s}$$

α_c_ is a function of α_0_ (a constant related to the properties of particles forming the cake) and the cake compressibility index (n) [15] as shown in Equation (5):

(5) $$\alpha_{c}=\alpha_{0}\Delta P^{n}$$

The closer the n value to 1, the higher the cake compressibility.

Moreover, Hermia dead-end blocking law models were used to differentiate between different fouling mechanisms via Equation (6) [15], where t is the filtration time (s), V is the water volume (L), k is the blocking law filtration coefficient, and n is the blocking law filtration exponent (unitless).

(6) $$\frac{d^{2}t}{dV^{2}}=k{\left(\frac{dt}{dV}\right)}^{n}$$

The linear expressions of the Hermia models are shown in Equations (7)–(10). To determine the main fouling mechanisms under the different filtration testing conditions, linear regression analysis was conducted, and the coefficients of the correlation (R^2^) values were estimated for the best-fitted model [15,16].

Complete pore blocking (n = 2): **ln J** = ln J_0_ + k_cb_ **t** (7)

Intermediate pore blocking (n = 1): **1/J** = 1/J_0_ + k_ib_ **t** (8)

Standard pore blocking (n = 1.5): **1/J^0.5^** = 1/J_0_^0.5^ + k_sb_ **t** (9)

Cake layer formation (n = 0.5): **1/J^2^** = 1/J_0_^2^ + k_cf_ **t** (10)

k_cb_, k_ib_, k_sb_, and k_cf_ represent the blocking law filtration coefficients for complete, intermediate, standard, and cake fouling, respectively [15,16].

In order to examine the effect of surface charge and the interaction between organic and inorganic colloids, the zeta potential was measured for individual and combined solutions using a Malvern ZetaSizer Nano. The molecular weight distribution of model foulants was measured using a UF fractionation method described by Kitis et al. [17] and a Malvern ZetaSizer Nano. In addition, three samples from the feed and permeate water were collected at different time intervals (after filtering with 100, 200, and 300 mL of feed water) and used for total organic carbon (TOC) analysis (using a Shimadzu TOC-VCPH/CPN analyzer) to determine the carbon rejection during each filtration experiment.

## 3. Results

### 3.1. The Impact of Inorganic Colloids on the Zeta Potential of BSA and SA

**Figure 2:**
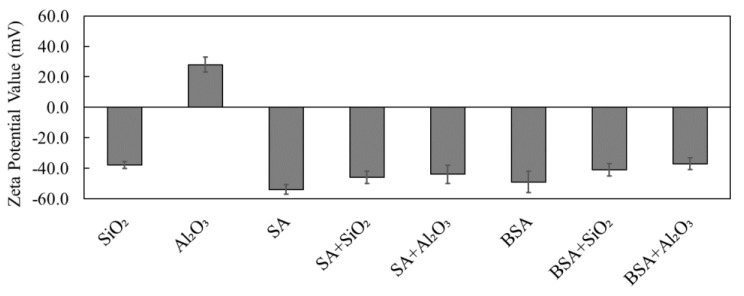
Measured zeta potential values of SiO_2_, Al_2_O_3_, SA, and BSA in single and combined solutions.

Figure 2 presents the measured surface zeta potentials of the individual and combined organic and inorganic solutions under the same solution pH conditions that were used in the experiments (pH of 7.0 ± 0.2). It can be clearly seen that for individual solutions, SiO_2_ had a negative zeta potential value of −36 ± 2.2 mV, while Al_2_O_3_ had a positive value of 28 ± 1.2 mV. On the other hand, the SA and BSA model organics had a negative charge of −55 ± 1.4 mV and −48 ± 3.3 mV, respectively. The zeta potential values reported here are in agreement with the results from a previous study [13], which reported that SiO_2_ and organics had a negative charge on their surfaces, whereas Al_2_O_3_ had a positive charge. When the combination of SiO_2_ and Al_2_O_3_ was added to the SA and BSA solutions, the zeta potential values of the combined solutions were higher than the zeta potentials of individual inorganics but lower than those of individual organic solutions, which were in the range between −40 mV and −45 mV. Moreover, it can be clearly seen that the combined organic and inorganic solutions all became negatively charged even though the zeta potential for Al_2_O_3_ was initially positive, demonstrating a stronger interaction between SA and BSA and Al_2_O_3_ in the combined solutions. Schulz et al. [13] and Taheri et al. [18] reported that organic substances could cause a surface coating to form on inorganic colloids, thus transforming their surface properties to a negative charge and reflecting the surface properties of organics. In addition, organic materials were reported to have a higher affinity to adsorb Al_2_O_3_ compared to SiO_2_ [13]. Thus, the findings here are consistent with those from other previous studies; however, the type of inorganic plays an important role in the zeta potential of SA and BSA.

### 3.2. Fouling of BSA and SA with Inorganics in Single and Combined Solutions

**Figure 3:**
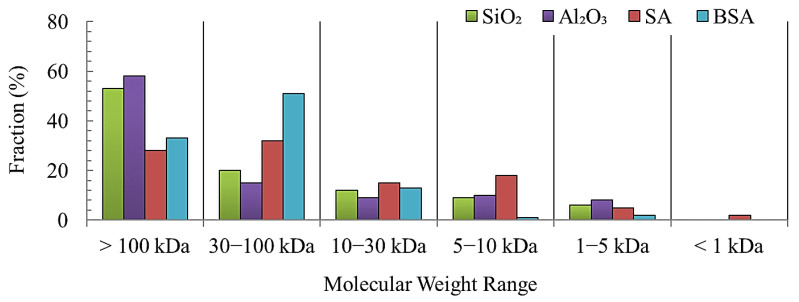
Molecular weight distribution of model foulants.

The molecular weight distribution (Figure 3) of model foulants was determined using the method detailed in Section 2.3. As can be seen, the SiO_2_ and Al_2_O_3_ colloids have a fairly similar size distribution in which the majority of particles (>55%) are larger than the MWCO of the UF membrane (100 KDa). The distribution of SA particles shows that 28% are larger than 100 KDa, and 35% are between 30 and 100 KDa. More than 30% of SA particles are smaller than 30 KDa. In contrast, 35% of BSA particles are larger than 100 KDa, whereas a larger percentage (>50%) of BSA particles are in the size range of 30–100 KDa. The differences in the size distribution of the model foulants are expected to influence their fouling and cleaning mechanisms.

**Figure 4:**
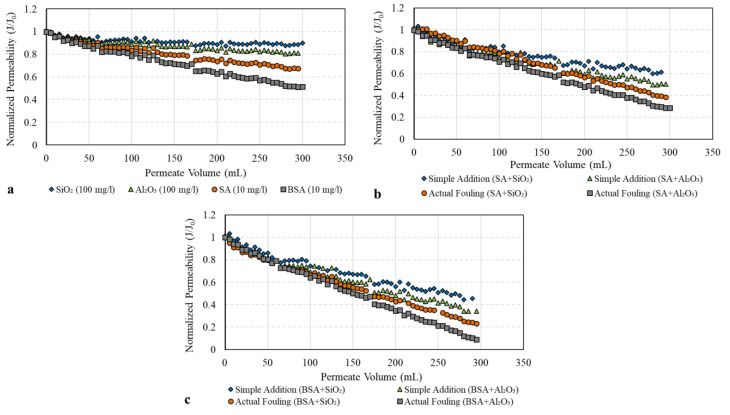
Membrane permeability during filtration of individual and combined organic and inorganic solutions: (**a**) fouling of individual solutions; (**b**) SA fouling *w*/*o* inorganic colloids; (**c**) BSA fouling *w*/*o* inorganic colloids.

To investigate the fouling behavior of SA and BSA with and without the addition of SiO_2_ and Al_2_O_3_ colloids, Figure 4 shows the decline in membrane permeability (represented by the normalized permeability, J/J_0_). From Figure 4a, it can be clearly seen that during the filtration of SiO_2_ only, the decline in normalized membrane permeability was minimal, reaching a value of 0.95. The filtration of Al_2_O_3_ alone, however, resulted in a greater permeability decline compared to SiO_2_, with a final value of 0.82. As the size distribution of both SiO_2_ and Al_2_O_3_ (Figure 3) is fairly similar, the difference in the filtration performance of the two colloids could be attributed to the differences in the surface charge as determined by the zeta potential measurements (refer to Figure 2). The Al_2_O_3_ surface was positively changed, indicating a higher affinity to adsorb on the surface that causes a greater flux decline. Meanwhile, the SiO_2_ surface was negatively charged, and thus, higher repulsion forces between SiO_2_ and the membrane were expected. Therefore, less adsorption and the permeability decline were caused by SiO_2_ colloids. Similar results were observed in previous studies [19,20,21] in which inorganic colloids alone contributed to a smaller permeability decline. The results of our study suggest that the zeta potential also influences the fouling behavior of the inorganic colloid; that is, a larger permeability decline was found with the positively charged Al_2_O_3_ surface compared to the negatively charged surface of SiO_2_.

Figure 4a also shows the decline in the membrane permeability during the filtration of individual SA and BSA solutions. The filtration of SA alone resulted in a noticeable decline in membrane permeability which reached a final value of 0.70; meanwhile, for BSA, permeability declined to 0.53 at the end of filtration. In this study, BSA had a slightly lower zeta potential value compared to SA (refer to Figure 2), indicating that BSA has a greater tendency to foul the membrane, causing a greater permeability decline compared to SA. Moreover, the majority of BSA particles were between 30 and 100 KDa (Figure 3), which is relatively smaller or closer to the MWCO of the UF membrane, thus resulting in the narrowing of the pores’ opening and the restriction of the flow through the membrane compared to SA particles. In addition, some BSA particles (>100 KDa) may have sealed the pores completely and contributed to a higher level of fouling.

Figure 4b,c illustrates the combined effect of organic and inorganic foulants on membrane permeability. For SA (Figure 4b), it can be seen that during the filtration of the combined SA and SiO_2_ solution, the permeability declined to 0.41, indicating much greater fouling compared to their individual model solutions, as observed in Figure 4a (final permeability of 0.70 for SA and 0.95 for SiO_2_). Moreover, the actual fouling of the combined SA and SiO_2_ solutions was higher than that found via the simple addition of their individual effect (final permeability of 0.65), as shown in Figure 4b. This fouling behavior was also observed during the filtration of SA and Al_2_O_3_ solutions. The presence of Al_2_O_3_ colloids during the filtration of SA resulted in a greater permeability decline compared to that with SiO_2_ (final permeability of 0.32). This could be related to the differences in the surface charge characteristics of Al_2_O_3_ and SiO_2_, in which Al_2_O_3_ with a positively charged surface increases the SA adsorption and causes a higher level of fouling compared to SiO_2_. The interaction effect of Al_2_O_3_ and SA was also noticeable, as the decline in membrane permeability was much higher than that found via the simple addition of their individual effects (Figure 4b).

Figure 4c shows the influence of SiO_2_ and Al_2_O_3_ on BSA fouling. The presence of SiO_2_ during the filtration of BSA increased membrane fouling with a final permeability of 0.22. Meanwhile, the combination of BSA and Al_2_O_3_ caused severe fouling of the UF membrane, resulting in a final permeability of 0.15. These results are consistent with that observed for SA, in which Al_2_O_3_ colloids have a much stronger influence on organic fouling compared to SiO_2_. Figure 4c also shows that the actual effect of the combined BSA and inorganic colloids on membrane permeability is higher than the value calculated with the simple addition of their individual effects. Moreover, BSA demonstrated a greater tendency to foul the UF membrane when combined with SiO_2_ and Al_2_O_3_ as compared to SA, indicating the influence of protein substances on membrane fouling and flux performance. Thus, the type of organics in water and their interaction with inorganics play an important role in the membrane fouling mechanism.

### 3.3. Effect of Inorganic Colloids on BSA and SA Rejection and Fouling Layer Properties

**Figure 5:**
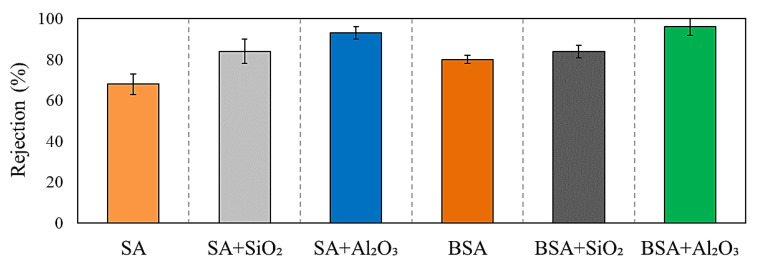
SA and BSA rejections in the presence and absence of SiO_2_ and Al_2_O_3_.

The rejection of SA and BSA with and without inorganic colloids was assessed and is presented in Figure 5. For individual organic solutions, the rejection of SA was 70 ± 4%; meanwhile, there was a higher rejection for BSA of 80 ± 3%. BSA had a higher percentage of particles close to or larger than the MWCO of the UF membrane (100 KDa), and thus, it had a higher retention compared to SA. On the other hand, SA has more than 45% of particles smaller than 100 KDa, which passed through the membrane to the permeate side, as illustrated in the retention values (Figure 5). The rejection trend of individual organic materials correlates well with the fouling tendency and permeability decline observed in Figure 4a, in which a higher rejection of BSA resulted in the highest fouling followed by SA. Figure 5 also shows the rejection percentages during the filtration of SA and BSA with inorganics in combined solutions. SiO_2_ and Al_2_O_3_ increased the rejection of SA to around 82 ± 3% and 91 ± 1%, respectively, indicating that aggregation of inorganic colloids with SA shifted the size of SA particles to a much larger size, which may have contributed to the increased rejection compared to SA alone. The rejection of BSA in the presence of SiO_2_ and Al_2_O_3_ also increased to 84 ± 2% and 95 ± 3%, respectively. This was also reflected in the higher level of fouling, and greater permeability decline observed during the filtration of individual Al_2_O_3_ and in combination with SA and BSA (refer to Figure 4).

**Figure 6:**
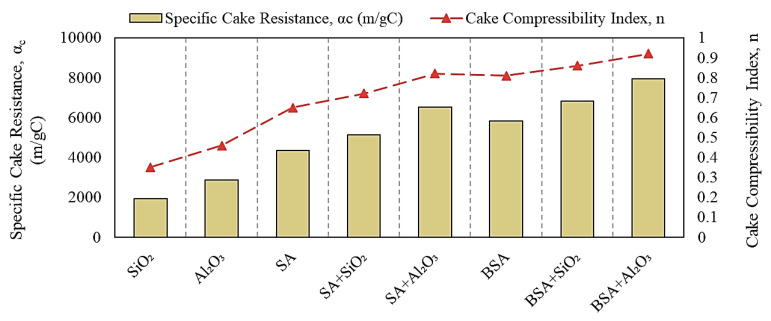
Specific cake resistance and compressibility index values.

To understand the effect of inorganic colloids on the fouling characteristics of SA and BSA, Figure 6 illustrates the α_c_ and n values calculated from Equations (4) and (5). For individual SiO_2_ and Al_2_O_3_ solutions, the specific cake resistances were 1.9 × 10^3^ and 2.8 × 10^3^ m/gC, respectively, with lower compressibility index values of 0.35 and 0.46, respectively. These results indicate that the fouling layer formed by SiO_2_ and Al_2_O_3_ was porous with an open structure, which resulted in less fouling and a smaller permeability decline, as reflected in Figure 4. The cake resistance formed by SA and BSA alone was 4.1 × 10^3^ and 5.8 × 10^3^ m/gC, respectively, both of which were highly compressible (n values of 0.65 and 0.81, respectively). The higher compressibility index values for SA and BSA indicate the formation of a less-permeable fouling layer on the membrane surface, which resulted in a larger permeability decline during the filtration of individual organic solutions. Filtration of the combined organic and inorganic solutions increased the specific resistance of the cake layer and resulted in a highly compressible fouling layer (n values range from 0.75 to 0.92), indicating that inorganic colloids changed the properties of the fouling formed by organic materials by increasing the cake resistance and decreasing the permeability of the fouling layer. Previous studies have reported that organic fouling can be enhanced in the co-presence of inorganic silica colloids [21,22,23]. In this study, although fouling increased with the addition of SiO_2_, which was in agreement with other studies, Al_2_O_3_ showed a higher effect on organic fouling compared to SiO_2_, which can be attributed to the positive zeta potential and the higher rejection of Al_2_O_3_, as observed in Figure 5. Moreover, the specific cake resistances formed in the presence of BSA were significantly higher compared to those formed with SA. Therefore, the more-compressible cake layer of BSA would impact both the membrane flux and cleaning efficacy (i.e., backwashing).

### 3.4. Analysis of BSA and SA Fouling Mechanisms with and without Inorganics

The results of the membrane permeability and fouling layer properties demonstrate the influence of inorganic colloids on SA and BSA fouling and rejection during filtration. The fouling models were fitted with the experimental data to determine the fouling mechanism for each condition. Table 2 illustrates the different models’ fitting of the experimental data and parameters of the linear regression during the filtration of individual and combined organic and inorganic materials. It can be observed that for the filtration of individual inorganic solutions, the cake filtration model has the highest R^2^ values of 0.88 and 0.82 for SiO_2_ and Al_2_O_3_, respectively. This indicates that the fouling of inorganic colloids occurred on the exterior surface of the membrane, thus leading to less pore blocking and a smaller permeability decline. This can be supported by the size distribution (Figure 3), which showed that the majority of SiO_2_ and Al_2_O_3_ particles are larger than the pore openings of the membrane (i.e., >100 KDa), and thus, external fouling occurred. On the other hand, for individual organic solutions, the standard pore-blocking model fits with the R^2^ value of 0.81 and 0.84 for SA and BSA, respectively, indicating that organic particles caused internal fouling and pore constriction and, thus, a larger permeability decline. Meng et al. [24] reported on the pore-blocking mechanism for alginate fouling, and their results were consistent with this study.

During the filtration of the combined SA and BSA solution with SiO_2_ and Al_2_O_3_, the fouling mechanism shifted to complete pore blocking with R^2^ values that ranged from 0.84 to 0.89, indicating an interaction effect between the organics and inorganics in water that resulted in sealing the membrane pores completely, and thus, a higher level of fouling. These results explain the significant increase in membrane fouling observed during the filtration of the combined model solutions. The occurrence of cake filtration for the combined model solutions is also possible (R^2^ range from 0.73 to 0.76), which implies that some membrane areas were covered by a cake layer which may have enhanced the membrane fouling in the present study.

### 3.5. Analysis of Fouling Resistances and Backwash Efficacy

**Figure 7:**
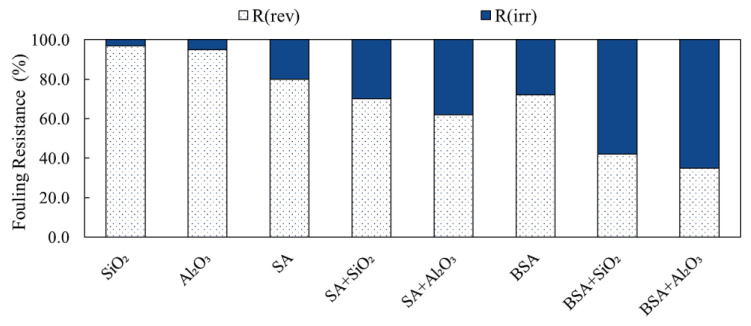
Fouling resistances and backwash efficacy of SA and BSA with and without inorganics.

SA and BSA fouling during filtration with and without inorganic colloids was categorized into reversible and irreversible fouling to assess backwash performance. Figure 7 presents the determined fouling resistances for the different filtration conditions. It is shown that during the filtration of individual SiO_2_ and Al_2_O_3_ solutions, the reversible fouling (R_rev_) was the highest and contributed to 95% and 92% of the total fouling resistance for SiO_2_ and Al_2_O_3_, respectively. This indicates that the fouling of individual inorganic colloids was mostly removable by backwash, which is reflected by the minimal decline in membrane permeability during filtration. Moreover, the fouling resistances correlate well with the cake formation fouling mechanism determined by the blocking law analysis (refer to Table 1). The irreversible fouling (R_irr_) of SiO_2_ and Al_2_O_3_ was negligible compared to the reversible portion, which contributed less than 10% of the total fouling resistance.

Unlike inorganic colloids, the R_rev_ of individual organic solutions was 76% and 68%, whereas the R_irr_ was 24% and 32% for SA and BSA, respectively. The irreversible fouling caused by organic materials indicates SA and BSA particles caused internal pore fouling, which resisted backwash. The flux decline caused by BSA, due to higher irreversibility as compared to SA, suggests that the type of organic constituents in water needs to be carefully considered when designing a membrane backwash procedure. Figure 6 also shows the changes in fouling resistances during the filtration of the combined organic and inorganic solutions. It can be clearly seen that for SA, the backwash efficacy of the membrane decreased to 68% and 60%, whereas greater irreversible fouling of 32% and 40% occurred when SiO_2_ and Al_2_O_3_ were present in water, respectively. For BSA, the backwash efficacy decreased significantly to 41% and 34% when SiO_2_ and Al_2_O_3_ were present in water, respectively. The observed decrease in the membrane backwash was due to the fact that the fouling of BSA and SA with inorganics in combined mixtures shifted to complete pore blocking (refer to Table 2), which resulted in the formation of a less-permeable fouling layer of higher specific resistance (refer to Figure 6), and hence, higher irreversible fouling. The changes in the fouling mechanism of the combined organic and inorganic solutions suggest that alterations to the membrane pretreatment and backwash strategies are recommended to ensure better control of membrane fouling and hence, improved permeability.

Table 3 presents a comparison of the findings of this study to those from previously published studies. As can be seen, previous studies reported a higher level of fouling and flux decline in UF and RO systems caused by the alginate and humic in the presence of inorganics (i.e., silica, Ca^2+^, etc.). The findings of this study demonstrated that the types of organics and inorganics in water play a crucial role in the membrane fouling mechanism and cleaning efficacy. That is, BSA caused more fouling compared to SA, while Al_2_O_3_ enhanced the fouling irreversibility of both types of organics, causing lower backwash efficacy. Thus, the membrane pretreatment and backwash strategies are recommended to be adjusted based on the type of organics in the water to ensure better control of membrane fouling and hence, improved permeability.

## 4. Conclusions

The fouling behavior of BSA and SA with SiO_2_ and Al_2_O_3_ in individual and combined solutions in a dead-end UF system was investigated. The key findings were:

- The individual presence of SiO_2_ or Al_2_O_3_ in water does not have a significant impact on UF fouling, as the decline in the normalized flux was minimal. However, Al_2_O_3_ has a higher affinity to adsorb on the membrane surface and causes a higher flux decline compared to SiO_2_.
- BSA filtration, with and without SiO_2_ and Al_2_O_3_, demonstrated a higher level of fouling and a greater permeability decline of the UF membrane compared to SA, resulting in a higher specific cake resistance and compressibility index. Moreover, backwashing was less effective with BSA compared to SA due to the higher irreversibility of BSA. Fouling of both BSA and SA was worsened with the addition of Al_2_O_3_.
- The fouling mechanism of BSA and SA shifted to complete pore blocking when they were combined with SiO_2_ and Al_2_O_3_, indicating an interaction effect between organics and inorganics in water that resulted in sealing the membrane pores completely, thus causing higher irreversible ratios and lower backwash efficacy.
- Our findings demonstrate that both the types of organics and inorganics in water influence UF membrane fouling and cleaning. Thus, it is suggested that both the pretreatment and cleaning of membrane systems need to be carefully designed and adjusted according to the types of organics and inorganics present in water. In addition, the effect of other types of inorganics (i.e., Mg^2+^, Ca^2+^, and Fe^2+^) in combined solutions on fouling and their cleaning methods requires future studies.

## Figures and Tables

**Table 1:** Feed water solutions used in fouling experiments.

Experiment	Model Foulant	Concentration	Ionic Strength
**1**	SiO_2_	100 mg/L	NaCl (2 mM) CaCl_2_ (1.5 mM)
**2**	Al_2_O_3_	100 mg/L	
**3**	SA	10 mg/L	
**4**	SA + SiO_2_	10 mg/L SA + 100 mg/L SiO_2_	
**5**	SA + Al_2_O_3_	10 mg/L SA + 100 mg/L Al_2_O_3_	
**6**	BSA	10 mg/L	
**7**	BSA + SiO_2_	10 mg/L BSA + 100 mg/L SiO_2_	
**8**	BSA + Al_2_O_3_	10 mg/L BSA + 100 mg/L Al_2_O_3_	

**Table 2:** Analysis of organic and inorganic fouling mechanisms (blocking law models).

Model Foulant	Complete Pore Blocking	Intermediate Pore Blocking	Standard Pore Blocking	Cake Layer Formation
	Coefficient of Correlation (R^2^)			
**SiO_2_**	0.56	0.42	0.64	0.88 *
**Al_2_O_3_**	0.41	0.55	0.66	0.82 *
**SA**	0.58	0.62	0.81 *	0.66
**SA + SiO_2_**	0.84 *	0.64	0.62	0.74
**SA + Al_2_O_3_**	0.88 *	0.68	0.69	0.73
**BSA**	0.68	0.62	0.84 *	0.63
**BSA + SiO_2_**	0.86 *	0.61	0.64	0.74
**BSA + Al_2_O_3_**	0.89 *	0.66	0.68	0.76

* represents best fit.

**Table 3:** Comparison of findings of this study to previous studies.

Reference	Membrane Type	Feed Water	Filtration Conditions	Fouling Results
Taheri et al. [18]	Regenerated cellulose UF	Humic acid and colloidal silica	Dead-end constant pressure	An increase in fouling was observed with increasing humic acid concentration in the mixture solution, whereas single solutions of humic acid or silica caused less fouling.
Wang at al. [21]	Thin-film composite RO	UF prefiltered secondary effluent	Cross-flow constant flux	Silica can be a significant component in membrane fouling in the co-presence of organic matter.
Xin et al. [3]	Flat-sheet MF	Alginate with Fe^2+^ and Ca^2+^	Dead-end constant pressure	Cake layer produced by the Fe^2+^-alginate had higher resistance than that produced by the Ca^2+^-alginate.
Wang et al. [2]	Flat-sheet RO	Seawater SA and sodium silicate	Cross-flow constant pressure	Higher fouling irreversibility with combined alginate and silicate solution compared to single solution.
Melián-Martel et al. [1]	Spiral-wound RO	Seawater SA and colloidal silica	Dead-end constant pressure	Higher flux decline with combined alginate and silica solution.
Zhang et al. [5]	PVDF UF	SA with Ca^2+^	Dead-end constant pressure	Change in alginate structure and increase in fouling formation was observed with increasing Ca^2+^ concentration in solution.
Kimura et al. [22]	Spiral-wound RO	MBR effluent	Cross-flow constant flux	Binding of divalent cations with functional groups of alginate forms a compact crosslinked gel layer, which results in severe membrane fouling.
Wang et al. [6]	PES UF	SA with Mg^2+^.	Cross-flow constant pressure	An increase in fouling and gel layer formation of alginate with increasing Mg^2+^ concentration in the solution.
This study	Regenerated cellulose UF	BSA and SA with and without Al_2_O_3_ and SiO_2_	Dead-end constant pressure	BSA resulted in greater flux decline and higher irreversibility compared to SA with or without SiO_2_ and Al_2_O_3_. BSA and SA fouling mechanism shifted from cake filtration to complete pore blocking when combined with inorganics. Backwashing was less effective with BSA compared to SA due to higher irreversibility of BSA. Fouling of both organics was worsened with the addition of Al_2_O_3_.

## Data Availability

All required data are included in the main manuscript.
